# Intestinal epithelial Gasdermin C is induced by IL-4R/STAT6 signaling but is dispensable for gut immune homeostasis

**DOI:** 10.1038/s41598-024-78336-z

**Published:** 2024-11-03

**Authors:** Reyes Gámez-Belmonte, Yara Wagner, Mousumi Mahapatro, Ru Wang, Lena Erkert, Miguel González-Acera, Roodline Cineus, Saskia Hainbuch, Jay V. Patankar, David Voehringer, Ahmed N. Hegazy, Markus F. Neurath, Stefan Wirtz, Christoph Becker

**Affiliations:** 1 Department of Medicine 1, Friedrich-Alexander-Universität Erlangen-Nürnberg (FAU) and Universitätsklinikum Erlangen, Erlangen, Germany; 2 Department of Gastroenterology, Infectiology and Rheumatology, Charité Universitätsmedizin, Berlin, Germany; 3 Deutsches Rheumaforschungszentrum Berlin (DRFZ), An Institute of the Leibniz Association, Berlin, Germany; 4 Institute of Immunology, Ludwig-Maximilians-Universität München, 80336 München, Germany; 5 Department of Infection Biology, University of Erlangen, 91054 Erlangen, Germany; 6 The Transregio 241 IBDome Consortium, Berlin, Germany; 7 Deutsches Zentrum Immuntherapie (DZI), Erlangen, Germany; 8 Department of Medicine 1, University Medical Centre Friedrich-Alexander-Universität Erlangen-Nürnberg, Erlangen, Germany

**Keywords:** Intestinal homeostasis, Gut pathology, Gasdermin, Cell biology, Gastroenterology

## Abstract

Gasdermin C is one of the least studied members of the gasdermin family of proteins, known for their critical involvement in pyroptosis and host defense. Furthermore, evidence for the role of Gasdermin C in the intestine is scarce and partly controversial. Here, we tested the functional role of Gasdermin C in intestinal homeostasis, inflammation and tumorigenesis. : We studied Gasdermin C in response to cytokines in intestinal organoids. We evaluated epithelial differentiation, cell death and immune infiltration under steady state conditions in a new mouse line deficient in Gasdermin C. The role of Gasdermin C was analyzed in acute colitis, infection and colitis-associated cancer. Gasdemin C is highly expressed in the intestinal epithelium and strongly induced by the type 2 cytokines IL-4 and IL-13 in a STAT6-dependent manner. Gasdermin C-deficient mice show no changes in tissue architecture and epithelial homeostasis. Epithelial organoids deficient in Gasdermin C develop normally and show no alterations in proliferation or cell death. No changes were found in models of acute colitis, type 2 intestinal infection and colitis-associated cancer. Gasdermin C genes are upregulated by type 2 immunity, yet appear dispensable for the development of intestinal inflammation, infection and colitis-associated cancer.

**Supplementary Information:**

The online version contains supplementary material available at 10.1038/s41598-024-78336-z.

## Introduction

The gasdermins (GSDM) are a superfamily of proteins that currently includes GSDMA, GSDMB, GSDMC, GSDMD, GSDME (also known as DFNA5) and PJVK (also known as DFNB59). Out of these, GSDMD is the best characterized member of the group. Several members of the GSDM family mediate a form of inflammatory cell death known as pyroptosis^1^. In its canonical form, pyroptotic death is mediated by inflammasome assembly, followed by the cleavage of GSDMD by activated Caspase-1. The N-terminal domain of cleaved GSDMD perforates the cell membrane, forming oligomeric pores. In addition to the secretion of IL-1β and IL-18, the formation of pores allows the entry of water, which causes cell swelling and osmotic lysis. With the exception of DFNB59, all the proteins in the GSDMs superfamily are characterized by an N-terminal pore-forming domain. However, pyroptosis is not the only cell death mechanism in which GSDMs are involved. In fact, during the last years, a number of reports have shown that GSDMs can execute other modes of programmed cell death, including apoptosis and NETosis^2^. Recently, a role of selected GSDMs in gut homeostasis and intestinal inflammation has also been reported. GSDMD is upregulated in IBD patients, however there are discrepancies regarding its protective or deleterious effect on intestinal inflammation. In one of these reports, it was shown that GSDMD-derived pores promotes colitis via IL-18 secretion from IECs^3^. In striking contrast, a different publication showed that in experimental colitis, GSDMD plays a protective role in macrophages but not in IECs^4^. Moreover, GSDME deficiency in nonhematopoietic cells alleviated experimental colitis in mice^5^. Despite belonging to the GSDM family, GSDMB seemingly plays a rather pyroptosis-independent role. Interestingly, GSDMB acts by promoting epithelial restitution and repair via FAK phosphorylation^6^.

In the gut, among the Gasdermin family, Gasdermin C (GSDMC) has only recently begun to attract attention. The role of this protein in health and disease is far from understood. It is noteworthy that, in contrast to the human GSDMC, the mouse genome contains four copies of the *Gsdmc* gene, arranged in tandem (*Gsdmc1-4*). Overall, the evidence for the role of GSDMC in the gut is scarce and, in some cases, controversial. Du et al., reported that N^6^-adenomethylation of *Gsdmc* is essential for the survival of mouse colonic Lgr5^+^IECs via a mechanism involving disruption of mitochondrial membrane potential and cytochrome c release. In support for this role, they also showed that GSDMC knockdown induces massive apoptosis in human colonic organoids. Hence, the authors propose that GSDMC functions as a critical mitochondrial stabilizer^7^. In contrast, no change in the stem cell compartment was reported by Zhao et al. Using mice with a specific deletion of *Gsdmc1-4* in IECs, the authors detected no differences in the transcriptome of these mice under steady state conditions, although they presented Gasdermin C as a driver of type 2 inflammation and a critical effector in the response to *Heligmosomoides polygyrus*helminth infection^8^. In conclusion, further work is needed to elucidate the role of GSDMs family in the gut.

In this study, we comprehensively analyze the role of GSDMC in the gut using newly generated GSDMC knockout mice and mouse models of intestinal inflammation, infection and tumorigenesis. We observed that Gasdermin C expression is highly induced by the type 2 cytokines IL-4 and IL-13 in a STAT6 dependent manner. Surprisingly, we observed that in our model, knockout of the four *Gsdmc1-4* genes did not result in any detectable phenotypes in homeostatic or pathological conditions that we tested.

## Results

### Gasdermin C isoforms are predominantly located in the gut

**Fig. 1:**
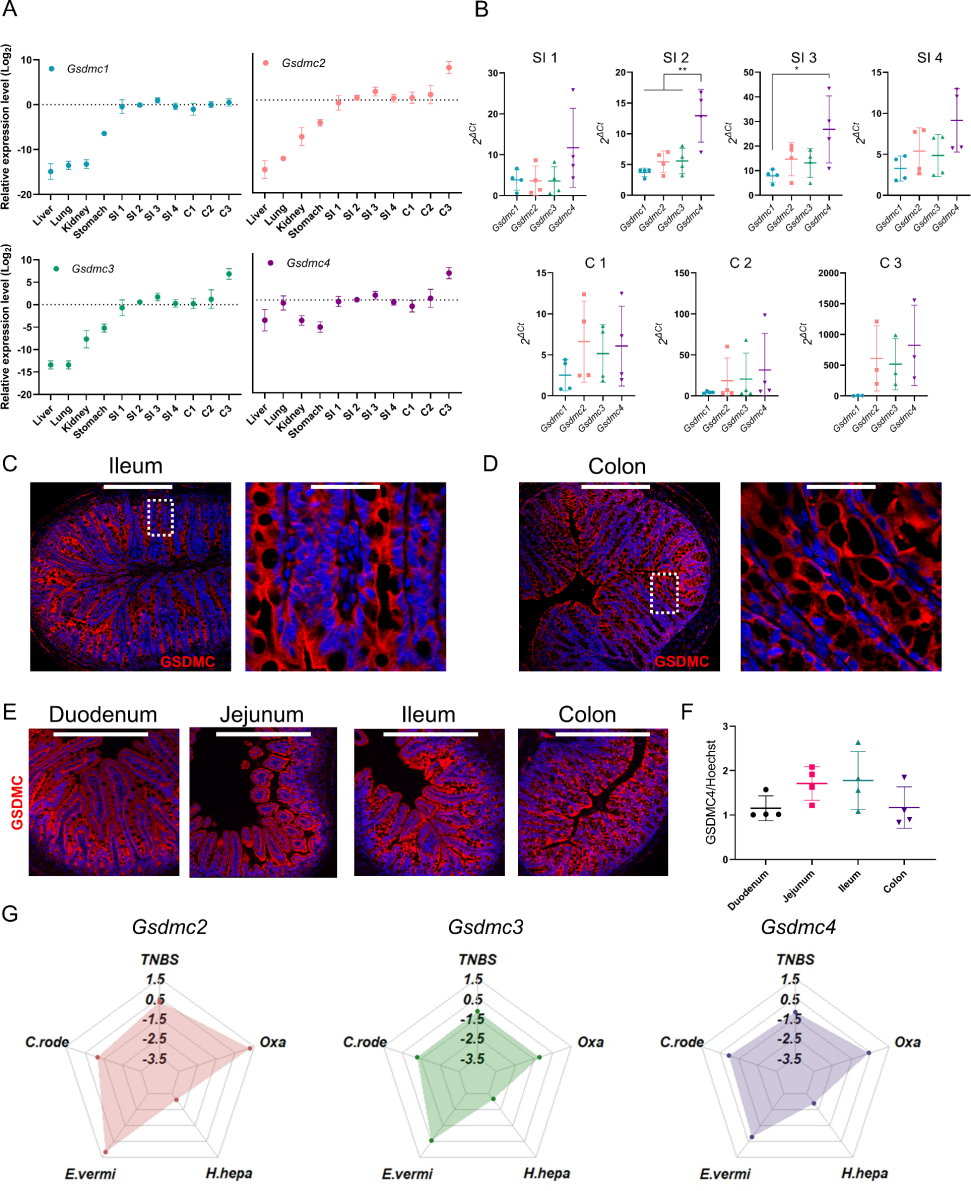
Gsmdc1-4 is highly expressed in gut tissue. (**A**) Relative mRNA levels of *Gsdmc1*, *Gsdmc2*, *Gsdmc3* and *Gsdmc4* along the gastrointestinal tract, liver, lung and kidney. (SI, small intestine; C, colon; 1–4 indicates proximal to distal). (**B**) Comparative expression of *Gsdmc1*, *Gsdmc2*, *Gsdmc3* and *Gsdmc4* along the gastrointestinal tract (SI, small intestine; C, colon). (**C**-**D**) Photomicrograph and insets of ileum and colon tissues stained for GSDMC4 (red). Counterstaining with Hoechst (blue). Scale bar 258.1 μm and 37.4 μm (HPV) (**E**) Representative images of GSDMC4-stained (red) gut tissues and (**F**) quantification. Counterstaining with Hoechst (blue). Scale bar 427.2 μm. (**G**) Spider plot representing changes in *Gsdmc2*, *Gsdmc3* and *Gsdmc4* expression in TNBS and Oxazolone (Oxa) inflammation models and in infection with Helicobacter hepaticus (H. hepa), Eimeria vermiformis (E.vermi) and Citrobacter rodentium (C.rode). *, and ** indicate the *p* < 0.05, < 0.01 respectively from One-Way ANOVA.

To characterize the role of the GSDMC family of proteins, we first examined the expression patterns of the different *Gsdmc* genes, including *Gsdmc1*, *Gsdmc2*,* Gsdmc3* and *Gsdmc4*, in different tissues (Fig. 1A). Surprisingly, we found that of all tested organs, the intestine exhibited the highest expression of all GSMDC isoforms. Of note, *Gsdmc1*, *Gsdmc2*, and *Gsdmc3* transcripts, were barely detected in tissues other than the intestine. All *Gsdmc* genes exhibit a similar expression along the gastrointestinal tract, with the exception of *Gsdmc4*, which shows higher levels of mRNA in proximal small intestinal segments (Fig. 1B). Concerning the precise cellular source, the analysis of a single-cell transcriptome of intestinal epithelial cells (IECs) revealed an uneven expression of the *Gsdmc1-4 *genes across the different IECs subsets (Supplementary Fig. 1A)^9^. In particular, *Gsdmc1*, *Gsdmc2*, *Gsdmc3*, and *Gsdmc4* exhibited the highest expression in the enterocyte subset, followed by the enterocyte progenitors. The presence of GSDMC in IECs was also supported by IHC performed on ileum and colon tissues, using a newly designed antibody raised against GSDMC4 (Fig. 1C-D). The procedure for the generation of this new antibody is depicted in Supplementary Fig. 1B. We next sought to assess GSDMC levels along the intestinal tract using this newly developed tool. We detected GSDMC4 in IECs across all sections of the intestine, showing consistent levels throughout (Fig. 1E-F). Having identified IECs as a niche for the expression of the *Gsdmc* family genes, we subsequently explored how their expression changes in pathological conditions. For this, we analyzed the expression pattern of the different *Gsdmc* genes (*Gsdmc1* was excluded due to low expression) in response to intestinal inflammation induced by chemical insults (TNBS, Oxazolone) or bacterial/apicomplexean infection (*Helicobacter hepaticus*, *Eimeria vermiformis*, *Citrobacter rodentium*) (Fig. 1G). A similar trend (downregulation) was observed for all *Gsdmc* genes in intestinal inflammation triggered by *C. rodentium* and *H. hepaticus* infection. In contrast, intestinal inflammation induced by TNBS instillation was not associated with significant changes in *Gsdmc1-4* expression. Notably, a distinct pattern emerged in oxazolone-mediated colitis and *E. vermiformis* infection. While *Gsdmc3* and *Gsdmc4* levels remained unchanged, *Gsdmc2* showed a marked upregulation. Collectively, these findings suggest both model-specific modulation of the *Gsdmc* genes and differences in the regulation of individual *Gsdmc* genes. Finally, given the preferential expression of the *Gsdmc1-4* in mature enterocytes and their progenitors, we hypothesized that strategies that alter the process of IEC differentiation may lead to changes in GSDMC expression. Treatment with DBZ, a known inhibitor of the γ-secretase complex, results in the inhibition of Notch signaling and, as a consequence, a marked imbalance in IEC differentiation toward the secretory lineage. As expected, in this context, a reduction in the absorptive lineage was associated with a dramatic decrease in all the GSDMC isoforms, observed at both the mRNA and the protein levels (Supplementary Fig. 2A-B).

### The basal expression of ***Gsdmc1-4 ***is driven by type 2 cytokine-induced- STAT6 signaling

**Fig. 2:**
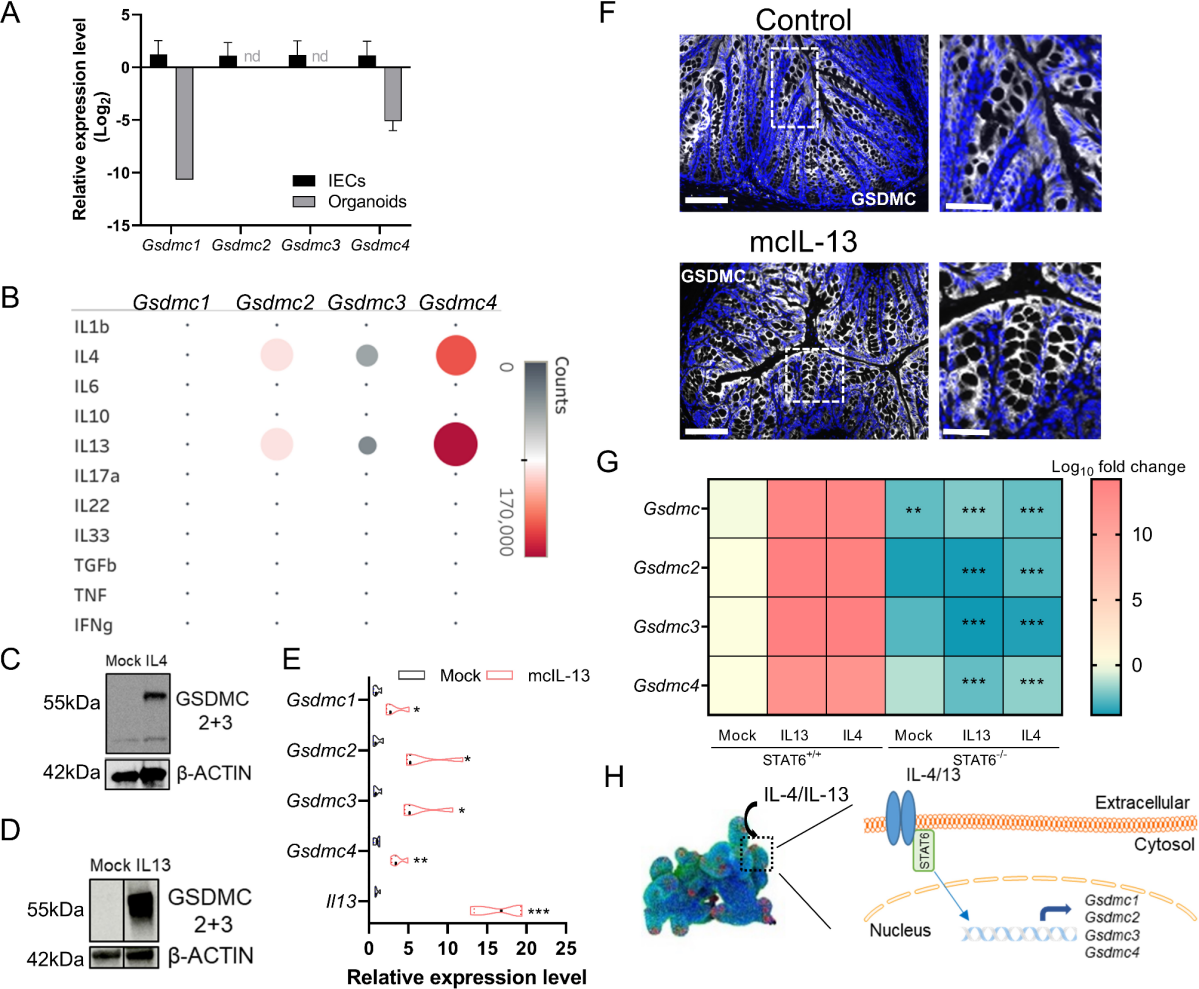
*Gsdmc1-4 * is upregulated *in vivo* and *in vitro* in response to Th2 cytokines. (**A**) Expression levels of *Gsdmc1-4* in freshly isolated IECs and in intestinal organoids. (**B**) *Gsdmc1*, *Gsdmc2*, *Gsdmc3* and *Gsdmc4* counts in intestinal organoids stimulated with 50 ng/ml of the indicated cytokines for 24 h. Level of GSDMC2 + 3 proteins in intestinal organoids stimulated with IL-4 (50 ng/ml, 24 h) (**C**) and IL-13 (**D**) (50 ng/ml, 24 h) (Western blot image represents cropped gel where irrelevant samples and lanes were removed for clarity. Original blots/gels are presented in Supplementary Fig. 4A-B). β-actin was used as a loading control. (**E**) Relative transcript levels of *Gsdmc1*, *Gsdmc2*, *Gsdmc3*, *Gsdmc4* and *Il13* in colon tissue from mock and mc-IL13 injected mice. (**F**) Photomicrographs and insets of mock and mc-IL13 injected mice stained for GSDMC4 (white) in colon tissue. Counterstaining with Hoechst (blue). Scale bar 100 μm and 50 μm (inset). (**G**) Heat map depicting *Gsdmc1*, *Gsdmc2*, *Gsdmc3* and *Gsdmc4* expression in *Stat6* proficient and deficient small intestinal organoids in response to IL-13 and IL-4 (50 ng/ml, 24 h). (**H**) Scheme of the modulation of *Gsdmc1-4* genes in response to type 2 cytokines. *, ** and *** indicate the *p* < 0.05, < 0.01 and < 0.001, respectively from Student’s t-test or Two-Way ANOVA.

Given the complexity of the changes we observed in *Gsdmc* gene expression in the different scenarios evaluated thus far, our subsequent efforts aimed at uncovering the factors responsible for mediating GSDMC modulation in IECs. For this, we cultured intestinal organoids and quantified the expression levels of *Gsdmc1-4* genes. Interestingly, while *Gsdmc1-4* were detected in freshly isolated IECs from the small intestine, the expression was barely detectable in small intestinal organoids cultured *in vitro* for 10 days (Fig. 2A). These data hinted at the potential requirement of soluble factors present in the intestinal milieu for the basal expression of *Gsdmc1-4*. In a previous report, *Gsdmc1-4* were identified as target genes of IL-4^10^. We extended this to test the ability of other cytokines to upregulate *Gsdmc1-4* expression. As depicted in Fig. 2B, of all cytokines tested, IL-4 and IL-13 markedly stimulated the expression of all *Gsdmc* isoforms. Western blotting confirmed the absence of GSDMC2 and GSDMC3 under steady state conditions and the remarkable induction of both proteins upon IL-4 and/or IL-13 stimulation in small intestinal organoids (Fig. 2C-D and Supplementary Fig. 4A-B). In line, this effect was also observed *in vivo*, after increasing the systemic abundance of IL-13 (Fig. 2E-F). Overexpression of IL-13 resulted in increased transcription of intestinal *Gsdmc1-4*, as shown by qPCR analysis for the different isoforms and immunostaining of GSDMC4 in the colon. Signal transducer and activator of transcription factor-6 (STAT6) is essential for mediating some of the effects induced by IL-4 and IL-13^11^. To prove the involvement of STAT6 in the effect induced by type 2 cytokines, we took advantage of small intestinal organoids generated from STAT6^−/−^ mice. In contrast to the expected upregulation of the *Gsdmc1-4* observed in STAT6 proficient organoids, the expression remained unaltered in STAT6 deficient organoids stimulated with IL-4 and IL-13 (Fig. 2G). In addition, basal expression of *Gsdmc1-4* was already reduced under unchallenged conditions. Altogether, our experiments identify IL-4 and IL-13 as the only cytokines tested, capable of modulating *Gsdmc1-4* expression via a STAT6-mediated mechanism (Fig. 2H).

### Mice lacking the expression of ***Gsdmc ***genes are viable and show no overt phenotype

**Fig. 3:**
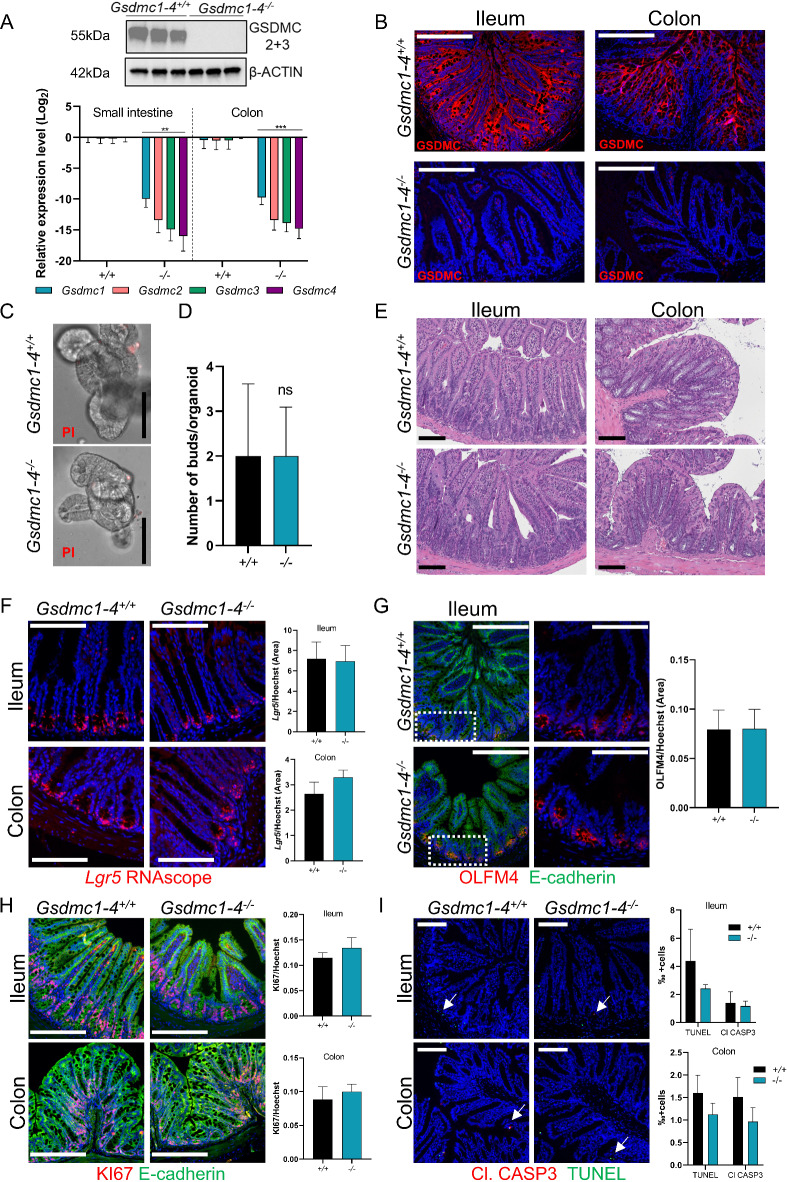
*Gsmdc1-4*^**−**/−^mice show no detectable alteration in steady state conditions. (**A**) GSDMC2 + 3 protein levels in the colon (top) and relative mRNA levels (bottom) of *Gsdmc1*, *Gsdmc2*, *Gsdmc3*, *Gsdmc4* in the small intestine and colon of *Gsdmc1-4*^+/+^ and *Gsdmc1-4*^−/−^ mice. β-actin was used as a loading control. Original blots/gels are presented in Supplementary Fig. 4C. (**B**) Representative images of GSDMC4-stained (red) ileum and colon from *Gsdmc1-4*^+/+^ and *Gsdmc1-4*^−/−^ mice. Counterstaining with Hoechst (blue). Scale bar 213.6 μm. (**C**) Intestinal organoids from *Gsdmc1-4*^+/+^ and *Gsdmc1-4*^−/−^ mice were treated with IL-13 (50 ng/ml) for 24 h and then stained with PI. Scale bar 100 μm. (**D**) Buds per organoid counted in small intestinal organoids isolated from *Gsdmc1-4*^+/+^ and *Gsdmc1-4*^−/−^ mice and stimulated with IL-13 (50 ng/ml, 24 h). (**E**) Examples of hematoxylin and eosin (H&E)-stained ileum and colon sections from *Gsdmc1-4*^+/+^ and *Gsdmc1-4*^−/−^ mice. Scale bar 100 μm. (**F**-**I**) Photomicrographs of ileum and colon sections from *Gsdmc1-4*^+/+^ and *Gsdmc1-4*^−/−^ mice stained for *Lgr5* RNA (**F**, scale bar 106 μm), OLFM4 and E-cadherin (**G**, scale bar 106 μm), KI67 and E-cadherin (**H**, 213.6 μm), or cleaved Caspase 3 and TUNEL assay (**I**, scale bar 100 μm) along the corresponding quantification. Counterstaining with Hoechst (blue). *n* = 5–6. ** and *** indicate the *p* < 0.001 and < 0.0001, respectively from Student’s t-test.

In order to determine the functional significance of th*e Gsdmc1-4* genes in intestinal homeostasis and disease, we generated a novel mouse line. Using CRISPR-Cas9 technology, a genomic region containing all 4 Gasdermin C genes were deleted to generate the *Gsdmc1-4*^*−/−*^ mice (Supplementary Fig. 2C). Deletion of the *Gsdmc1-4* was confirmed by western blotting, qPCR (Fig. 3A and Supplementary Fig. 4C) and immunostaining (Fig. 3B) in both small intestine and colon tissues. After confirming the successful deletion of all *Gsdmc* genes, we next aimed to determine the functional role of the *Gsdmc* genes in IECs, using small intestinal organoids derived from *Gsdmc1-4*^*+/+*^ and *Gsdmc1-4*^*−/−*^ mice. As mentioned before (Fig. 2A), the expression of *Gsdmc1-4* is profoundly diminished in cultured IECs, likely due to the absence of factors produced by non-epithelial cells and present in the intestinal microenvironment. To achieve a high expression of *Gsdmc1-4 in vitro*, we stimulated the organoids with IL-13 and analyzed the effects of the deletion of *Gsdmc1-4*. Neither *Gsdmc1-4*^*+/+*^ nor *Gsdmc1-4*^*−/−*^ organoids showed significant cell death in response to IL-13 stimulation, as measured by propidium iodide binding to DNA (Fig. 3C). Next, the effect of *Gsmdc1-4* deletion on organoid development was assessed. The organoids developed a similar size and comparable number of buds were counted in organoids derived from *Gsdmc1-4*^*+/+*^ and *Gsdmc1-4*^*−/−*^ mice (Fig. 3D). Moreover, and in agreement with a normal development, in the presence of IL-13, no differences were detected in EdU incorporation, a surrogate marker for cell proliferation; as well as in Olfactomedin 4 (OLFM4) and ULEX immunostaining, reflecting normal stem and goblet cell proportions, respectively (Supplementary Fig. 2D-E). To explore if *Gsdmc1-4* deletion is associated with more subtle changes, we performed bulk RNA sequencing of intestinal organoids derived from *Gsdmc1-4*^*+/+*^ and *Gsdmc1-4*^*−/−*^ mice. However, ablation of *Gsdmc1-4* did not result in significant changes in the transcriptome of IECs (Supplementary Fig. 2F). Collectively, our data reveal that Gasdermin C is dispensable for intestinal organoid growth and differentiation.

We then investigated if GSDMC is involved in the interplay between the epithelial and non-epithelial compartments in intestinal homeostasis *in vivo*. Histological analysis of the small intestine and colon tissues revealed no visible alterations in tissue morphology (Fig. 3E). In agreement with the normal differentiation and development of organoids, stem cell population and proliferation as defined by *Lgr5* expression, OLFM4 and KI67 stainings showed comparable patterns between *Gsdmc1-4*^*+/+*^ and *Gsdmc1-4*^*−/−*^ mice (Fig. 3F-H). To further evaluate cell differentiation, we immunostained for Mucin2 (MUC2) and Doublecortin Like Kinase 1 (DCLK1), markers of goblet and tuft cells, that is, secretory cell subsets in ileum and colon tissues. The deletion of the *Gsdmc* genes did not affect the differentiation of the IECs toward the secretory lineage *in vivo* (Supplementary Fig. 2G-H). In addition to the epithelium, we studied the impact of GSDMC on the gut immune compartment. CD45^+^ cell content in the ileum and colon was indistinguishable from control mice (Supplementary Fig. 2I-J). Due to the known involvement of the Gasdermin family in cell death and immune homeostasis, we determined the presence of cell death in small intestine and colon tissues from *Gsdmc1-4*^*+/+*^ and *Gsdmc1-4*^*−/−*^ mice. However, in unchallenged mice, apoptotic cells (positive for cleaved Caspase-3 and TUNEL) and presumed non-apoptotic cell death (only TUNEL positive) were scarcely detected, independently of the genotype (Fig. 3I).

## The GSDMC proteins play no major role in the context of intestinal inflammation and healing

**Fig. 4:**
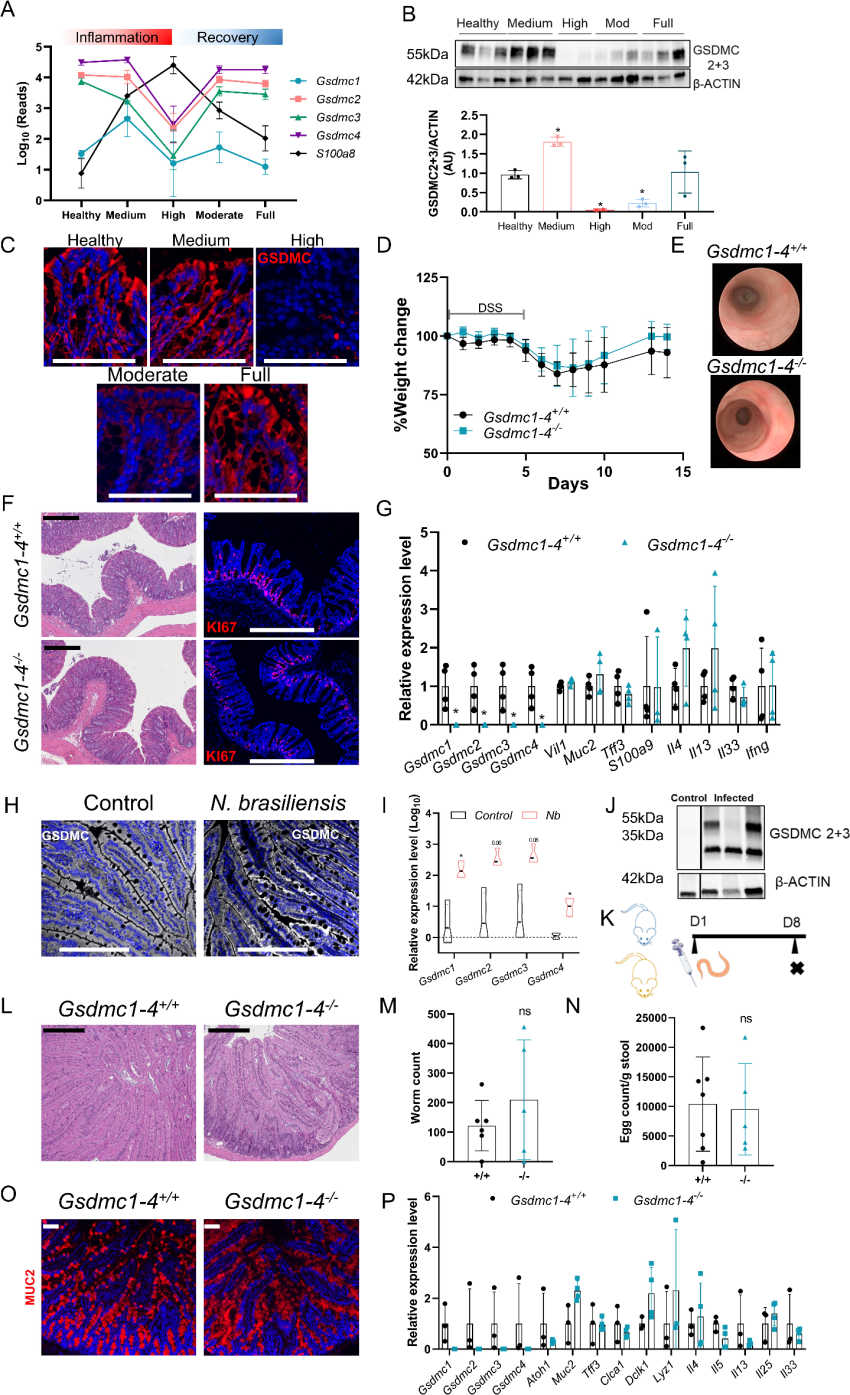
*Gsdmc1-4 *deletion does not affect resolution of inflammation in DSS colitis or worm expulsion after infection with *Nippostrongylus brasiliensis*. (**A**) Expression of *Gsdmc1*, *Gsdmc2*, *Gsdmc3*, *Gsdmc4* and *S100a8* during the different stages of colitis (publicly available, E-MTAB-9850^23^. (**B**) Protein and mRNA levels of GSDMC1-4 during the course of DSS-induced colitis. β-actin was used as a loading control. Original blots/gels are presented in Supplementary Fig. 4D. (**C**) Representative immunofluorescence images of GSDMC4 (red) in different stages of colitis. Counterstaining with Hoechst (blue). Scale bar 100 μm. (**D**) Relative percent change in body weight and (**E**) representative colonoscopy images of *Gsdmc1-4*^+/+^ and *Gsdmc1-4*^−/−^ mice subjected to DSS. (**F**) Representative images of H&E and KI67-stained colon sections at the end of the experiment. Counterstaining with Hoechst (blue). Scale bar 250 and 427 μm. (**G**) Relative mRNA levels of the indicated genes in colon samples from *Gsdmc1-4*^+/+^ and *Gsdmc1-4*^−/−^ mice at the end of the experiment (*n* = 4 per group). The DSS experiment shown is representative of three independent replicates. (**H**) Representative images of GSDMC4-stained (white) duodenum sections from *N. brasiliensis* infected and control mice (*n* = 3 per group). Counterstaining with Hoechst (blue). Scale bar 213.6 μm. (**I**) Relative expression levels of *Gsdmc1*, *Gsdmc2*, *Gsdmc3* and *Gsdmc4* in duodenum samples from control and *N. brasiliensis*-infected mice (*n* = 4 per group). (**J**) GSDMC2 + 3 protein levels in duodenum from control and *N. brasiliensis*-infected animals. β-actin was used as a loading control (Western blot image represents cropped gel where irrelevant samples and lanes were removed for clarity. Original blots/gels are presented in Supplementary Fig. 4E). (**K**) Scheme showing the experimental protocol for the infection with *N. brasiliensis*. (**L**) Representative H&E staining of duodenal sections (scale bar 250 μm) from *Gsdmc1-4*^+/+^ and *Gsdmc1-4*^−/−^ mice infected with *N. brasiliensis*. Worm (**M**) and egg (**N**) counts measured in tissue and feces, respectively, from *N. brasiliensis*-infected *Gsdmc1-4*^+/+^ and *Gsdmc1-4*^−/−^ mice (*Gsdmc1-4*^+/+^*n* = 7 and *Gsdmc1-4*^−/−^*n* = 5). (**O**) Photomicrograph of duodenal sections stained for MUC2 (red) from *Gsdmc1-4* proficient and deficient mice infected with *N. brasiliensis*. Counterstaining with Hoechst (blue). Scale bar 100 μm. (**P**) Relative mRNA levels of indicated genes in duodenum samples from *N. brasiliensis*-infected *Gsdmc1-4*^+/+^ and *Gsdmc1-4*^−/−^ mice (*Gsdmc1-4*^+/+^*n* = 3 and *Gsdmc1-4*^−/−^*n* = 4). The *N. brasiliensis* infection experiment displayed is representative of three independent experiments *, indicates the *p* < 0.05, ns = not significant from Student’s t-test.

To uncover the role of the *Gsdmc* family genes under pathological conditions, we firstly assessed the effect of *Gsdmc1-4* gene deletion on intestinal inflammation induced by dextran sulfate sodium (DSS). Interestingly, the levels of intestinal G*sdmc1-4* transcripts in wildtype mice are altered during the course of DSS-induced intestinal inflammation (Fig. 4A-C). In particular, the levels of all the *Gsdmc* genes were drastically reduced in highly inflamed tissue. In addition, *Gsdmc1-4* reached levels comparable to healthy tissue only after the full recovery following the cessation of DSS administration in drinking water (Fig. 4B-C and Supplementary Fig. 4D). Given the dynamic expression of *Gsdmc1-4* during intestinal inflammation and healing, we challenged *Gsdmc1-4*^*+/+*^ and *Gsdmc1-4*^*−/−*^ mice with DSS for 5 days followed by a recovery phase of 10 days. Both body weight measurements and endoscopic evaluation revealed comparable levels of inflammation and healing progression (Fig. 4D-E). In support of the macroscopic observation, the comparison of both groups by histology and KI67 staining, a surrogate marker of proliferation, yielded no differences (Fig. 4F) and neither did the analysis of inflammatory and IECs markers in colon tissue (Fig. 4G). These results show that intestinal inflammation induced by DSS and the subsequent healing process are not affected by the expression of *Gsdmc1-4*.

### GSDMC is nonessential for ***N. brasiliensis ***clearance

Gasdermin C has been implicated in type 2 immune responses against helminthic parasites^8^. Infection with the nematode *N.brasiliensis *induces a pronounced type 2 response, characterized by massive expansion of Th2 cells in the lung and the small intestine. IL-4 and IL-13, produced by ILC2s, play a critical role for timely worm expulsion and tissue repair^12^. Our own experiments suggest that *Gsdmc1-4* are target genes of Th2 cytokines and, in addition, a recent publication describes the *Gsdmc* family of genes as an important effector of anti-helminth immunity against another parasite, the helminth *Heligmosomoides polygyrus*^8^ *.* Similar to *H. polygyrus*, *N. brasiliensis* infection induced a marked upregulation of the *Gsdmc1-4* transcripts (Fig. 4H-I). Furthermore, similar to *H. polygyrus* infection, the increased expression occurred in parallel with the detection of the cleaved form of GSDMC (Fig. 4J and Supplementary Fig. 4E). These data prompted us to investigate whether *Gsdmc* family genes might be important mediators in the response against the nematode *N. brasiliensis* (Fig. 4K). However, 8 days post *N. brasiliensis*-infection, microscopic damage and worm expulsion, determined by counting adult worms in the small intestine, were comparable in *Gsdmc1-4*^*+/+*^ and *Gsdmc1-4*^*−/−*^ mice (Fig. 4L-M). In addition, both mouse strains had similar numbers of *N. brasiliensis* eggs in their feces (Fig. 4N). Nematode infection induces goblet cell hyperplasia and augmented mucus secretion^13^. To assess both outcomes provoked by the nematode infection, we analyzed goblet cells and mucus production in infected *Gsdmc1-4*^*+/+*^ and *Gsdmc1-4*^*−/−*^ mice. However, MUC2 production and goblet cell markers were comparable between *Gsdmc1-4*^*+/+*^ and *Gsdmc1-4*^*−/−*^ mice (Fig. 4O-P). Similarly, the mRNA levels of the type 2 cytokines (*Il4*,* Il5* and *Il13*), and the alarmins *Il25* and *Il33* were similar (Fig. 4P). Overall, our experiment revealed that GSDMC is dispensable for the inflammatory response and worm expulsion during *N. brasiliensis* infection.

### The ***Gsdmc ***family of genes are dispensable in an experimental model of colitis-associated colorectal cancer

Finally, we explored the impact of modulating the *Gsdmc* genes in the AOM-DSS model, the standard animal model for colitis-associated colorectal cancer. We immunostained for GSDMC4 in AOM-DSS-induced colon tumors. Interestingly, while GSDMC4 was present in IECs in normal mucosa, its expression was notably diminished in tumor tissue (Supplementary Fig. 3A). Accordingly, western blotting confirmed the downregulation of GSDMC2 and GSDMC3 in tumors compared to normal mucosa (Supplementary Fig. 3B and Supplementary Fig. 4F). To investigate the functional involvement of GSDMC in intestinal tumorigenesis, we utilized the AOM-DSS model of colitis-associated colorectal cancer in *Gsdmc1-4*^*+/+*^ and *Gsdmc1-4*^*−/−*^ mice. As observed in acute DSS colitis, body weight was comparable in response to DSS administration (Supplementary Fig. 3C). At the end of the experiment, tumor number and size (Supplementary Fig. 3D-G) were measured at the macroscopic and microscopic level. The analysis of these parameters showed no differences between *Gsdmc1-4*^*+/+*^ and *Gsdmc1-4*^*−/−*^ mice. Altogether, our experiment reveals that GSDMC does not play a predominant role in inflammation-dependent intestinal tumorigenesis.

## Discussion

In recent years, a growing interest in the GSDMs family of proteins has led to a remarkable progress in the mechanistical understanding of pyroptotic cell death and the discovery of novel physiological functions of their cleaved forms. In our study, we show that the *Gsdmc* family genes are predominantly expressed along the intestine. In order to study the role of GSDMCs in intestinal homeostasis and disease, we have developed a new mouse line that is deficient in all *Gsdmc* family genes.

Although there has been increasing interest in GSDMC in recent years, the current scientific literature on the role of GSDMC in the gut is scarce, inconclusive and even partly contradictory. Notably, there are striking discrepancies regarding the role of GSDMC in cell death and survival, both in steady state and during intestinal tumorigenesis. A recent publication showed that GSDMC knockdown in human colonic organoids led to impaired organoid growth and massive apoptosis. Detailed analysis of the organoids revealed an abolished LGR5 expression and the induction of pro-apoptotic pathways. Underscoring the importance of GSDMC in the gut epithelium, the authors described how GSDMC is required for Lgr5^+^stem cell survival and colon homeostasis^7^. In striking contrast, Zhao et al., reported no alterations in the intestinal epithelium of GSDMC-deficient mice^8^. The results of our own mouse model are consistent with the latter report, as no evidence of increased cell death was observed under steady state conditions. Furthermore, our own data revealed no effect of GSDMC for organoid development or epithelial cell death *in vitro*. This discrepancy may highlight differences between human and mouse GSDMC. In fact, while there is only one *GSDMC* gene in humans, the mouse genome contains four different *Gsdmc* genes^14^. However, the differences in regulation and function between human and mouse GSDMC require further investigation. In close association with its role during cell death, the available evidence points to a controversial effect of GSDMC in intestinal tumorigenesis. Interestingly, GSDMC is upregulated in mouse and human colorectal cancer^15^. Using cancer cell lines, including colon cancer, Zhang et al., reported that GSDMC-induced pyroptosis mediates the antitumor effect of α-Ketoglutarate, an essential metabolite in the tricarboxylic acid cycle^16^. Along the same lines, Xi et al., described that the overexpression of *Gsdmc2* in HEK293 cells induced robust pyroptosis. Interestingly, the enhanced lytic cell death was exclusive to *Gsdmc2*, as the other GSDMC tested, *Gsdmc4*, was ineffective^10^. Surprisingly however, not only did Miguchi et al., report the opposite effect, that is, no GSDMC-mediated cell death, they also observed that silencing *GSDMC *in human colorectal cancer cell lines significantly reduced cell proliferation *in vitro* and in tumor growth in a xenograft model *in vivo*^15^. Accordingly, the authors suggested that GSDMC functions as an oncogene, rather than a cell death inducer, suggesting it as a promising therapeutic target^15^. Using an established model of colorectal cancer in mice, namely colitis-associated colorectal cancer (AOM-DSS), we observed further discrepancies with the aforementioned reports. First, we detected a marked downregulation of all *Gsdmc* genes in intestinal tumorigenesis. In addition, and in contrast to the reported effect in human cell lines, the deletion of the *Gsdmc1-4* did not alter tumor initiation or development in our experiments. Thus, our studies in this model do not support an important role for GSDMC in proliferation or cell death *in vivo* or in other mechanisms that alter tumor growth. The AOM-DSS model of colorectal cancer offers the advantage of recapitulating the tumor microenvironment and immune interactions more accurately than xenograft models or *in vitro* models. Collectively, no clear picture has emerged so far of whether GSDMC might promote or suppress cancer development. In view of the conflicting results, it is tempting to speculate that several factors, such as the tissue and cell type investigated, the species (human vs. mouse) or tumor stage make it difficult to draw conclusions about the role of GSDMC in cancer. In the specific case of colorectal cancer, we cannot exclude the possibility that GSDMC only exerts a tumor-promoting effect in advanced stages.

GSDMC has been reported to be involved in type 2 immune responses and intestinal infection and inflammation. As shown by others^8,10^ and our own research, *Gsdmc1-4* are target genes of the type 2 cytokines IL-4 and IL-13 via STAT6 dependent activation. Type 2 cytokines play a key role in regulating immunity against helminth parasites. During parasitic infection, IL-4 and IL-13 induce the *Gsdmc1-4 *expression in the intestine. It has been hypothesized that cleaved GSDMC forms pores in IECs through which cytokines and antiparasitic factors are released^8,10^. Consistent with the role of GSDMC in anti-helminth response, one study reported that the specific ablation of *Gsdmc1-4* genes in intestinal epithelial cells resulted in increased worm burden after infection with *Heligmosomoides polygyrus*. The mechanism proposed in this publication involved the GSDMC pores in the unconventional secretion of IL-33. This effect appeared to be specific to parasitic infection, as the course of acute intestinal inflammation induced by DSS was not affected^8,17^. Consistent with this, no differences in DSS-induced colitis were observed in our newly developed mouse line deficient in GSDMC. However, in sharp contrast to the aforementioned study, our own studies using the type 2 helminth infection model *Nippostrongylus brasiliensis*, found no changes in the levels of inflammation, IEC markers, mucus production and worm burden in GSDMC-deficient mice. Thus, GSDMC does not seem to be essential for type 2 driven anti helminth immunity. Both parasites differ in their life cycle and, more importantly, in the duration of infection and induction of the granuloma response. While *H. polygyrus* establishes a stable, long-lasting infection with a strong intestinal granuloma response, *N. brasiliensis *infection involves extraintestinal stages and resembles an acute infection that develops without intestinal granuloma formation^18^. Whether GSDMC is only relevant in chronic and more dramatic phenotypes remains to be investigated. Collectively, our data confirm that GSDMC in mice is strongly induced by the type 2 cytokines IL-4 and IL-13 via STAT6 signaling. However, our data show that GSDMC is dispensable for immune homeostasis of the gut in steady state and also in models of intestinal infection, inflammation and cancer development, despite being strongly expressed in the intestinal epithelium *in vivo*. In agreement with the data from Zhao et al., our data do not support an overt effect of GSDMC on epithelial homeostasis, contradicting previous reports by Du et al., In conclusion, the field of GSDMC research is still in its infancy and the differences in the results obtained suggest a complex regulation of GSDMC, with a number of factors influencing their functions. Further investigations are warranted to help clarify the nature of GSDMC regulation and function in the intestine and related diseases.

## Methods

### Mice

*Gsdmc1-4*^*−/−*^ mice (B6-*Gsdmc1-4*-^tm1Agb^/J) were generated using CRISPR/Cas9 technology to introduce Cas9/guide RNA (gRNA) ribonucleoproteins into mouse embryos (Applied Stem Cell). Only heterozygous mice were used for breeding. Males and females littermates were used in all the experiments. Mice were maintained in individually ventilated cages under conditions of consistent humidity (40–60%) and temperature (18–23 °C), with an equal light: dark cycle (12 h), and had *ad libitum* access to rodent chow diet and drinking water. In the dextran sulfate sodium (DSS) experiment, mice were given 2.5% DSS in drinking water for 5 days, followed by a 10 day recovery period. Mini-endoscopy was performed to assess the recovery after DSS challenge.

The γ-secretase-specific inhibitor DBZ was administered intraperitoneally (i.p.) at a concentration of 30 µmol/kg/day, diluted in 0.5% hydroxyethylcellulose, for 7 consecutive days, as described before^19^. Wildtype animals used in this study were procured from Janvier Labs. Mice were sacrificed on day 8 after the first dose.

*Nippostrongylus brasiliensis* L3 were recovered from the faeces of infected rats, mixed with activated charcoal, and cultured in humidified chambers at room temperature. After extensive washing in sterile 0.9% saline (37 °C), 500 larvae were injected subcutaneously into mice in 200 µl of saline. Mice were provided with water containing antibiotics (Borgal 24%, Virbac) for the first 7 days.

In the Azoxymethane (AOM)/DSS model, *Gsdmc1-4*^+/+^ and *Gsdmc1-4*^-/-^mice were injected i.p. once with AOM (10 mg/kg, Sigma-Aldrich) followed by three cycles of DSS (1.5% (w/v)) for 5 days, with an interval of two weeks in between. Mini-colonoscopy was performed and tumor number and size (mm) were analyzed in the colon and categorized as previously described^20^.

*In vivo* expression of IL-13 was performed as described previously^21^. Wildtype animals used in this study were procured from Janvier Labs. Mice were sacrificed twelve days after vector administration.

The institutional review board and the ethics committee of the University of Erlangen-Nürnberg and the ethics commission of Lower Franconia approved animal experiments. All experiments were performed in accordance with relevant guidelines and regulations and ARRIVE guidelines. Mice were euthanised by cervical dislocation under isoflurane anaesthesia.

### IECs isolation

Small intestines were dissected, cut longitudinally and divided into small fragments. The fragments were incubated at 37 °C with 2mM EDTA. After 15 min, the fragments were passed through a 70 μm mesh filter and the supernatant obtained was centrifuged. The recovered cells were used for RNA preparation.

## Small intestinal organoids

Small intestinal organoids were generated as previously described^22^. In brief, intestinal crypts were isolated from mouse small intestine and resuspended in Matrigel (Corning). After solidification of the 3D-dome, cells were cultured in Advanced DMEM/F12 (Thermo Fisher Scientific) supplemented with glutamine (2 mM, Invitrogen), HEPES (10mM, Sigma-Aldrich), Penicillin-Streptomycin (100 U/mL and 100 µg/mL), R-spondin and Noggin conditioned medium, B27 (5x, Gibco), N-acetylcystein ( 1mM, Sigma-Aldrich), Primocin (100 µg/mL, Invivogen) and EGF (50 ng/mL, Immunotools).

Organoids from wildtype (WT) and STAT6^−/−^ mice were stimulated with IL-13 or IL-4 (50 ng/ml, Peprotech) for 24 h. WT organoids were stimulated with IL-1β (50 ng/ml, Immunotools), IL-4 (50 ng/ml, Peprotech), IL-6 (50 ng/ml, Immunotools), IL-10 (50 ng/ml, Biolegend), IL-13 (50 ng/ml, Peprotech), IL-17A (50 ng/ml, Immunotools), IL-22 (100 ng/ml, Invitrogen), IL-33 (50 ng/ml, Biolegend), TGF-β (50 ng/ml, Immunotools), TNF (50 ng/mL, Immunotools) and IFN-γ (50 ng/ml, eBioscence) for 24 h.

## Immunohistochemistry (IHC)

Formalin-fixed paraffin-embedded tissue sections were deparaffinized. Heat-induced antigen retrieval was performed in Tris-EDTA buffer, tissue sections were incubated with the following primary antibodies: GSDMC4 (1:200, GeneScript), KI67 (1:200, ab16667, Abcam), MUC2 (1:200, NBP1-31231, Novus), DCLK1 (1:200, ab31704, Abcam), OLFM4 (1:200, 39141, CST), F4/80 (1:200, 70076, CST), Cleaved Caspase-3 (1:200, 9661, CST). CD4 staining was performed on cryosections (1:200, 553043, BD Bioscience). TUNEL staining was performed using the In Situ Cell Death Detection Kit, TMR red (Roche). Nuclei were counterstained with Hoechst 33342 (1:1000, Invitrogen). Small intestinal organoids were fixed in 4% PFA, permeabilized with 0.1% Triton X-100 and then incubated with *Ulex Europaeus* Agglutinin I (UEA-1; 1:750, FL-1061, Vector) or the primary antibody OLFM4. Nuclei were counterstained with Hoechst 33342. Incorporation of EdU into DNA in tumor organoids was measured using EdU Proliferation Kit (iFluor 488, Abcam). Immunofluorescence images were acquired using the Leica DMI6000 B inverted fluorescence microscope (Leica Microsystems) or the Leica laser-scanning confocal microscope.

### Gene expression analysis

Total RNA was extracted from small intestinal organoids, tissue and IECs using a RNA isolation kit (NucleoSpin kit, Macherey Nagel), following the manufacter’s protocol. cDNA was obtained by reverse transcription using SCRIPT cDNA Synthesis Kit (Jena Bioscience). Real-time PCR was performed using specific QIAGEN QuantiTect Primer Assays. For normalisation, *Hprt* was used as a housekeeping gene.

### Transcriptome meta-analysis

For analyzing the expression of *Gsdmc1-4* and *S100a8* in the different stages of colitis we used the European Bioinformatics Insitute ArrayExpress, through which the publicly available dataset E-MTAB-9850 was obtained.

### mRNA sequencing

Following RNA extraction and quality assessment, the samples underwent sequencing on an Illumina Novaseq 6000 platform, producing paired-end reads. The sequences were then aligned to the reference genome using STAR (version 2.7.0d) and quantified with featureCounts (version 1.6.4). Differential expression analysis between sample groups was conducted with DESeq2 (version 1.24.0). Further analyses, including enrichment and clustering, were carried out using bioinformatics tools.

### Western blot

Tissue and small intestinal organoids were homogenized in Lysis buffer: M-PER for organoids; and T-PER for tissue (Thermo Scientific), with Pierce protease and phosphatase inhibitor Mini Tablets (Thermo Scientific) and Phenylmethylsulfonyl fluoride (PMSF, Roche) in both cases. Homogenates were centrifuged at 14000*g* for 20 min at 4 ⁰C. Protein concentration was determined by Bradford assay. Samples were boiled for 5 min in LDS sample buffer 4x (Invitrogen), separated by SDS–PAGE using a MiniProtean-TGX gel (4–15% polyacrylamide; Bio-Rad), blotted onto nitrocellulose membranes (Bio-Rad), and probed with the following antibodies: GSDMC2 + 3 (1:1000, ab229896, Abcam), β-actin-HRP (1:10000, ab49900, Abcam), and GAPDH (1:5000, 2118, CST). Blots were incubated with the HRP-conjugated anti-rabbit secondary antibody.

### Statistical analysis

Data were analyzed by Student’s t test, One-Way ANOVA and Two-Way ANOVA using GraphPad Prism. Significance levels are indicated as *p < 0.05, **p < 0.01 and ***p < 0.001. All data are presented as mean values ± SD.

## Electronic supplementary material

Below is the link to the electronic supplementary material.

Supplementary Material 1

Supplementary Material 2

Supplementary Material 3

Supplementary Material 4

Supplementary Material 5

## Data Availability

Data are available in a public, open access repository. Data are available on reasonable request. The publicly available datasets used in this study are published in Array Express service of the Molecular Biology Laboratory–European Bioinformatics Institute under accession number: E-MTAB-9850 (https://www.ebi.ac.uk/biostudies/arrayexpress/studies/E-MTAB-9850?query=%20E-MTAB-9850).
