# Supplementary material for: Intestinal epithelial Gasdermin C is induced by IL-4R/STAT6 signaling but is dispensable for gut immune homeostasis
Source: Sci Rep. 2024 Nov 3;14:26522. doi: 10.1038/s41598-024-78336-z (PMC11532336; doi:10.1038/s41598-024-78336-z)
Supplement: Supplementary file 1 — Supplementary Material 1 [file 41598_2024_78336_MOESM1_ESM.docx]

**SUPPLEMENTARY FIGURE LEGENDS**

**Supplementary Figure 1: *Gsdmc1-4* expression in IEC subsets and scheme of the development of a new antibody against mouse GSDMC4. (A)** *Gsdmc1*, *Gsdmc2*, *Gsdmc3* and *Gsdmc4* normalized counts in intestinal epithelial cell subsets from publicly available scRNA-Seq data^1^. (B) Once the protein has been synthesized, mice are immunised. The first step is to screen the animals' serum for antibodies to GSDMC4. In the next step, antibody-producing B cells are fused with myeloma cells. Finally, antibodies are purified from selected hybrids and tested in wild-type and *Gsdmc1-4*^-/-^ mice.

**Supplementary Figure 2: Gasdermin C is dispensable for gut and IEC homeostasis**. (A) Relative expression levels of *Gsdmc1*, *Gsdmc2*, *Gsdmc3* and *Gsdmc4* in intestinal tissues from wildtype mice treated with DBZ or vehicle for 7 days (30 µmol/kg/day). (B) GSDMC4 protein immunostained (white) in small intestinal tissue from control and DBZ-treated mice. Counterstaining with Hoechst (blue). Scale bar 213.6 µm (C) Schematic representation of the generation of the *Gsdmc1-4^-/-^* mice using CRISPR/Cas9 technology. (D-E) Small intestinal organoids from *Gsdmc1-4*^+/+^ and *Gsdmc1-4*^-/-^ treated with IL-13 (50 ng/ml, 24h) immunostained for OLFM4, EdU (D) and ULEX (E), along the corresponding quantification. Counterstaining with Hoechst (blue). Scale bar 100 µm. (F) Volcano plot from an RNA-seq experiment performed in small intestinal organoids of *Gsdmc1-4*^-/-^ versus *Gsdmc1-4*^+/+^ mice (the dashed horizontal line signals statistical significance threshold). (G-J) Photomicrographs of ileum and colon sections from *Gsdmc1-4*^+/+^ and *Gsdmc1-4*^-/-^ mice stained for MUC2 (red) and E-cadherin (green) (G), DCLK1 (red) and E-cadherin (green) (H), and CD45 in the Ileum (I) and the colon (J) along the corresponding quantification. Counterstaining with Hoechst (blue). Scale bar 213.6 µm and 100 µm (CD45). *, indicates the p <0.05, ns = not significant from Student’s t-test.

**Supplementary Figure 3: GSDMC is not involved in colitis-associated colorectal cancer**. (A) Representative images and insets of GSDMC4-stained tumor colon sections (right panel shows higher magnification) sections from mice treated with AOM-DSS (N = normal epithelium; T = tumor cells). Tumors are marked by a dashed line. Counterstaining with Hoechst (blue). Scale bar 427.2 µm, inset 213.6 µm (B) Levels of GSDMC2 and GSDMC3 proteins in normal mucosa and tumors induced by AOM-DSS treatment were measured by western blotting. Original blots/gels are presented in Supplementary Figure 4F. (C) Relative percent change in body weight of *Gsdmc1-4^+/+^* and *Gsdmc1-4^-/-^* mice subjected to AOM-DSS treatment (*Gsdmc1-4*^+/+^ n=5 and *Gsdmc1-4*^-/-^ n=4). (D) Representative colonoscopy images of *Gsdmc1-4*^+/+^ and *Gsdmc1-4*^-/-^ mice subjected to AOM-DSS treatment. (E) Tumor number and (F) tumor size classification measured in *Gsdmc1-4*^+/+^ and *Gsdmc1-4*^-/-^ mice subjected to AOM-DSS treatment. (G) Examples of conventional H&E histology of tumor sections from *Gsdmc1-4*^+/+^ and *Gsdmc1-4*^-/-^ mice induced by AOM-DSS treatment. Scale bar 500 µm. The AOM/DSS experiment shown is representative of three independent experiments. **, indicates the p <0.01 from Student’s t-test.

**Supplementary Figure 4: Original blots from western blotting images in Figure 2C (A), Figure 2D (B), Figure 3A (C), Figure 4B (D), Figure 4J (E) and Supplementary Figure 3B (F).**

1 Haber, A. L. *et al.* A single-cell survey of the small intestinal epithelium. *Nature* **551**, 333-339, doi:10.1038/nature24489 (2017).
